# Comparison of Sociodemographic and Nutritional Characteristics between Self-Reported Vegetarians, Vegans, and Meat-Eaters from the NutriNet-Santé Study

**DOI:** 10.3390/nu9091023

**Published:** 2017-09-15

**Authors:** Benjamin Allès, Julia Baudry, Caroline Méjean, Mathilde Touvier, Sandrine Péneau, Serge Hercberg, Emmanuelle Kesse-Guyot

**Affiliations:** 1 Equipe de Recherche en Epidémiologie Nutritionnelle (EREN), Centre de Recherche en Epidémiologie et Statistiques (CRESS), Université Paris 13, Inserm (U1153), Inra (U1125), Cnam, COMUE Sorbonne Paris Cité, F-93017 Bobigny, France; j.baudry@eren.smbh.univ-paris13.fr (J.B.); m.touvier@eren.smbh.univ-paris13.fr (M.T.); s.peneau@eren.smbh.univ-paris13.fr (S.P.); s.hercberg@eren.smbh.univ-paris13.fr (S.H.); e.kesse@eren.smbh.univ-paris13.fr (E.K.-G.); 2 Institut National de la Recherche Agronomique, INRA, UMR 1110 MOISA, F-34000 Montpellier, France; caroline.mejean@inra.fr; 3 Department of Public Health, Avicenne Hospital, F-93300 Bobigny, France

**Keywords:** vegetarians, vegans, diet, sociodemographic characteristics, dietary nutrient inadequacy

## Abstract

Background: There is a growing trend for vegetarian and vegan diets in many Western countries. Epidemiological evidence suggesting that such diets may help in maintaining good health is rising. However, dietary and sociodemographic characteristics of vegetarians and vegans are not well known. The aim of this cross-sectional study was to describe sociodemographic and nutritional characteristics of self-reported, adult vegetarians and vegans, compared to meat-eaters, from the French NutriNet-Santé study. Methods: Participants were asked if they were following a specific diet. They were then classified into three self-reported diet groups: 90,664 meat-eaters, 2370 vegetarians, and 789 vegans. Dietary data were collected using three repeated 24-h dietary records. Multivariable polytomic logistic regression models were perfomed to assess the association between the sociodemographic characteristics and type of diet. The prevalence of nutrient intake inadequacy was estimated, by sex and age for micronutrients, as well as by type of self-reported diet. Results: Compared with meat-eaters, vegetarians were more likely to have a higher educational level, whereas vegans had a lower education level. Compared with meat-eaters, vegetarians were more likely to be women, younger individuals, and to be self-employed or never employed rather than managerial staff. Vegetarians and vegans substituted animal protein-dense products with a higher consumption of plant protein-dense products (e.g., soy-based products or legumes). Vegetarians had the most balanced diets in terms of macronutrients, but also had a better adherence to French dietary guidelines. Vegetarians exhibited a lower estimated prevalence of inadequacies for micronutrients such as antioxidant vitamins (e.g., for vitamin E, 28.9% for vegetarian women <55 years of age vs. 41.6% in meat-eaters) while vegans exhibited a higher estimated prevalence of inadequacies for some nutrients, in particular vitamin B12 (69.9% in men and 83.4% in women <55 years of age), compared to meat-eaters. Conclusions: Our study highlighted that, overall, self-reported vegetarians and vegans may meet nutritional recommendations.

## 1. Introduction

Vegetarian diets can be defined as the partial exclusion of animal products (with the systematic exclusion of red meat and poultry, and some other animal products such as fish, eggs, and/or dairy products, depending on the type of vegetarianism [1]), whereas a vegan diet implies the complete exclusion of animal products (including all animal-based products such as added fats) [2]. In Western countries, the estimated prevalence of individuals following these diets varies between 1% and 10%. These estimates do not come from national or official observational surveys, but from polls and surveys conducted directly by vegetarian societies [3]. There is a growing trend for vegetarian and vegan diets in France, as in many other Western countries [1,4]; however, little is known about the sociodemographic and nutritional characteristics of such consumers.

A few studies, mostly conducted in Northern Europe or North America, have focused on the sociodemographic and lifestyle characteristics of vegetarians and vegans [5,6,7,8,9]. Most of those studies have shown that vegetarians are more likely to belong to higher socioeconomic categories compared to meat-eaters. They also have healthier lifestyles (e.g., lower prevalence of smokers).

Epidemiological evidence concerning the potential impact of vegetarian diets on health is rising. In Western countries, it has been reported that vegetarians compared to non-vegetarians had a lower mortality from ischemic heart disease, whereas no significant association with all-cause mortality has been reported [10]. Vegetarian diets may also be beneficial against diabetes [11], cancer risk [10], diverticular disease [12], some ocular diseases such as cataract [13], hypertension [14], and kidney stones [15]. However, it has been suggested that such diets could also be associated with a lower bone density and a higher risk of fracture [16,17], especially in vegans. The underlying mechanisms relating vegetarianism and health may involve adequate and inadequate intake of critical nutrients [2,18]. 

Only a few observational cohort studies have described in the same sample nutrient profiles, nutrient intake inadequacy, and the compliance with nutritional recommendations among vegetarians [6,19,20,21,22]. These studies reported that vegetarians had adequate intake of nutrients involved in the prevention of several chronic diseases such as polyunsaturated fatty acids, dietary fibers, and antioxidants. Conversely, diets excluding most animal products may result in deficiencies in macronutrients such as n-3 fatty acids, as well as micronutrients such as vitamin B12, vitamin D, and iodine [6,19,23].

Few studies focusing on vegetarian and vegan diets have described food sources of nutrients [24,25]. For example, vegetarians and vegans may adopt strategies to substitute animal protein-dense foods with plant protein-dense foods, thus covering their protein requirement. These strategies would result in increased consumption of meat substitute foods (e.g., tofu, processed textured soy protein food) [25]. Despite the fact that these products are more and more present in the food market [4], no recent data is available on the consumption of meat substitutes by vegetarians or vegans. 

The aim of the present cross-sectional study was to investigate sociodemographic and nutritional characteristics, such as the prevalence of dietary nutrient inadequacy, related to self-reported vegetarianism or veganism in French adults. Another aim of this study was to describe the intake of plant-based protein-dense foods and other plant-based food products that have been more recently introduced in the French food market among self-reported vegetarians, vegans, and meat-eaters.

## 2. Material and Methods

The study sample was composed of participants in the NutriNet-Santé Study, a large web-based prospective observational cohort launched in France in May 2009. Participants were recruited among Internet-using adults from the general population aged 18 years or older (>80% of the French adult population) [26]. The study was designed to investigate determinants of dietary behaviors and nutritional status as well as the relationships between nutrition and health. The design, methods, and rationale of this study have been previously described [26]. Briefly, participants had to fill in an initial set of questionnaires assessing sociodemographic, lifestyle, physical activity, anthropometry, and dietary factors, along with health status, to be included in the cohort. These baseline questionnaires were tested against traditional assessment methods (paper or interview by a dietitian) [27,28,29,30]. Each month, they were invited to fill out other optional questionnaires related to determinants of food behavior and various nutritional and health-related topics. 

This study was conducted according to guidelines laid down in the Declaration of Helsinki, and all procedures were approved by the Institutional Review Board of the French Institute for Health and Medical Research (IRB Inserm No. 0000388FWA00005831) and the Commission Nationale Informatique et Libertés (CNIL No. 908450 and No. 909216). Electronic informed consent to participate in the study was obtained from all subjects. 

### 2.1. Data Collection

#### 2.1.1. Definition of Vegetarian Diets and Dietary Intake Assessment 

At baseline, participants were asked whether they were following a vegetarian diet corresponding to the exclusion of some animal products, or a vegan diet corresponding to the complete exclusion of animal products. The question was as follows: “Currently, do you follow a specific diet?” yes/no. If yes, participants had to specify the main reason: “for medical reasons (other than weight loss); weight loss; to avoid gaining weight; to stay in shape; because I am a vegetarian (I do not eat meat but I eat other animal products); because I am a vegan (I do not eat any meat, nor fish, nor eggs, nor dairy products); because of personal or religious beliefs”. Based on this question, participants were classified into three groups: vegetarians, vegans, or meat-eaters (if they did not declare following vegetarian or vegan diets). We used this self-reported classification in order to account for the actual perception of the participants. Beyond consumption, this may reflect social requirement or lack of specific vegetarian food products in nutritional databases. Explanatory text was used in order to improve the accuracy of this self-report.

Dietary data were collected using web-based, self-administered 24-h dietary records via an interactive interface. At enrollment and yearly thereafter, participants were invited to provide three 24-h records (during one weekend day and two weekdays) [26]. These records were randomly assigned over a two-week period. The web-based dietary assessment method relies on a meal-based approach, recording all foods and beverages (type and quantity) consumed at breakfast, lunch, dinner, and all other eating occasions. First, participants filled in the names of all food items eaten, and then they estimated portion sizes for each reported food and beverage item according to standard measurements (e.g., home containers, grams displayed on the package) or using photographs available via the interactive interface. These photographs, based on a validated picture booklet [30], represented more than 250 foods (corresponding to 1000 generic foods) in seven different portion sizes. The accuracy and validity of web-based 24-h dietary records have been assessed by comparison to interviews by trained dietitians [29] and against 24-h urinary biomarkers [31,32]. 

Participants in our sample were included if they had completed the three 24-h dietary records at baseline. For each participant, daily mean food group consumptions (in grams) were calculated from 24-h records, weighted according to the day (week or weekend). Daily intakes for energy, macro-, and micronutrients were estimated using a published composition database [33] completed for recent market foods and recipes. Energy under-reporters were identified by the method proposed by Black [34]. 

Twenty-nine food groups were created, (i) 22 groups gathering foods classified according to nutritional considerations with sufficient details to be compared to the French Nutritional guidelines (PNNS) [35,36]: fruit, vegetables, legumes, potatoes and other tubers, whole starchy foods (whole pasta, whole bread, etc.), refined cereals and starchy foods (pasta or bread made with refined flour, etc.), uncooked cereals and seeds (oatmeal, sesame seeds, flaxseed, etc.), meat, poultry, fish and other seafood, eggs, processed meat, offal, dairy products, animal added fats, oils, salty snacks and biscuits, sweet and fatty foods (pastries, biscuits, cookies, chocolate, honey, jam and spreads), sugary drinks, sugar-free drinks, alcoholic beverages; and (ii) seven groups gathering foods that represented plant-based products and meat substitutes emerging on food markets as plant-sourced protein-dense products: quinoa, corn and other cereals, nuts and seeds, textured soy proteins products, vegetarian patties, germinated seeds, cookies and French diet or “digestive” biscuits enriched with cereals, and rice crackers. These seven food groups were created by gathering items separated from the French dietary guidelines food groups to better assess whether vegetarians or vegans had different intakes of these emerging foods (on the French market) compared to meat-eaters.

#### 2.1.2. Sociodemographic and Lifestyle Factors

Socioeconomic and demographic variables included sex, age, marital status, household composition, having at least one child or not, place of residence, education, occupation, household, living area, income, and smoking, which were all collected at baseline. Educational level was classified into four categories: primary education, secondary education, higher education (corresponding to at least three years after bachelor), and not answered. Participants were asked for their monthly household income including salary, social benefits, family allowance, and income (optional question). The household income per month was calculated by consumption units (CU) [37]. Categories used for monthly income were the following: <1200 €, 1200–1800 €, 1800–2700 €, and >2700 € per CU, as well as a category for individuals who refused to answer.

Household composition was classified into four classes: single without children, two adults living as a couple without children, and two adults living as a couple with children. Living area was coded based on zip codes into eight groups according to the French census data: Paris area, East-Center, East, Mediterranean area, North, West, Paris basin area, and South-West. Body Mass Index (BMI) (in kg/m^2^) was computed as the ratio of weight to squared height and then classified following the WHO guidelines [38]. Smoking status was classified into three categories: current smoker, former smoker, or never smoked.

### 2.2. Statistical Analysis

The present analyses focused on participants included in the NutriNet-Santé cohort study since May 2009, living in the French metropolitan area, who had complete and valid dietary intake data for our study. They had completed at least three 24-h dietary records at baseline and were not energy under-reporters, did not have missing sociodemographic data or missing data regarding the practice of a diet such as a vegetarian or vegan diet. Women declaring to be pregnant at their inclusion were also excluded because they may have unusual dietary intakes.

A univariable description of the individual characteristics according to the types of diet was performed. Then, multivariable polytomic logistitic regression models were perfomed to assess the association between sociodemographic characteristics and the type of diet using meat-eaters as a reference. 

The mean daily intakes of 29 food groups were adjusted for sex, age, and total energy intake. 

Dietary micronutrient intakes were adjusted for sex, age, and total energy intake using a method based on the residual method [39]. The contribution of macronutrients (in %) were adjusted for sex and age. Comparisons of macronutrients, micronutrients, and energy intakes between vegetarians, vegans, and meat-eaters were carried out using analysis of covariance (ANCOVA) tests. Compliance with French nutritional recommendations for macronutrients was assessed by computing the percentage of energy intake from carbohydrates, lipids, and proteins, as well as fiber intake in grams. The prevalence of individuals respecting French recommendations for macronutrient intake was then assessed (acceptable distribution range for proteins: 10–20% below 70 years of age, 15–20% above 70 years of age; for lipids: 35–40%, for carbohydrates: 40–55% and fibers ≥30 g/day, for individuals both below and above 70 years of age) [40]. Compliance with French nutritional recommendations for micronutrients was assessed by computing the prevalence of dietary nutrient inadequacy [41]. The measurement error model proposed by the National Research Council [42] and developed by Nusser et al. [43] was applied to the observed daily dietary intake, in order to remove the effects of day-to-day intake variability [44]. The proportion of subjects with reported intake below the estimated average requirement was estimated for each nutrient by sex and age category, following French nutritional recommendations [45]. It was established that, at the population level, this proportion represents an unbiased estimate of the proportion of subjects whose intake is below their respective requirements, also called ‘prevalence of dietary nutrient inadequacy’ [41]. Descriptions of corresponding mean daily micronutrient intakes by age and sex groups following French nutritional recommendations were also conducted.

Finally, the modified Programme National Nutrition Santé Guideline Score (mPNNS-GS), an a priori dietary index, was also computed as an indicator of the adherence to French dietary guidelines [46]. The maximum score is 13.5, reflecting the highest level of adherence to the French dietary guidelines. Mean scores of mPNNS-GS were computed for each diet groups adjusted for age and sex. 

All statistical analyses were performed using SAS software (version 9.4, SAS Institute Inc., Cary, NS, USA).

## 3. Results

The overall final sample included 93,823 participants; among them there was a large majority of women (78%), and the mean age was 48.7 years (SD = 14.7) (Table 1). The study sample included 2370 vegetarians, 789 vegans (3.4% of individuals within the sample declared themselves vegetarians or vegans), and 90,664 meat-eaters. Nearly 65% of the sample had an educational level higher than high school. Twenty-three percent of the participants were managerial staff and nearly 20% were manual workers. Seventeen percent of individuals had a low household income and 73% were living as a couple with or without children. Nearly 30% of the sample were overweight or obese (BMI > 25 kg/m^2^).

### 3.1. Sociodemographic and Individual Characteristics

Compared with meat-eaters, vegetarians were more likely to be women, younger individuals, to be self-employed or never employed rather than managerial staff, to belong to lower income groups, to be single without children, and to have a BMI < 20 (Table 2). Vegans were more likely to have a lower educational level and to be men.

### 3.2. Plant-Based and Animal-Based Products Intake

Overall, self-reported vegetarians and vegans had a higher consumption of plant-based products compared to meat-eaters (Table 3). Moreover, vegetarians had the highest consumption of eggs, sweet and fatty foods (equally with meat-eaters), and a lower consumption of seafood and animal added fats. Vegans had the highest consumption of animal protein substitutes, including textured soy products, vegetarian patties, as well as soy, almond, and rice milk, and other plant-based beverages. Furthermore, they had the lowest intakes of non-fatty cereals and the highest consumption of uncooked cereals and seeds, as well as whole starchy foods (whole pasta, bread, etc.). Along with the highest consumption of animal-based products (except eggs), meat-eaters also showed the highest intakes of unrefined cereals and starchy food, salty snacks and biscuits, sweet and fatty foods, soft drinks, and alcoholic beverages.

Vegetarians had the highest mean mPNNS-GS compared to meat-eaters, while vegans had the lowest mean mPNNS-GS (Table 3).

### 3.3. Compliance with Recommendations for Macronutrients

The fiber intake of vegans and vegetarians was 75% and 33% higher than that of meat-eaters, respectively (Table 4). Meat-eaters also had the highest alcohol intake (Table 4). Vegans had the lowest dietary intake of vitamin D (Table 4). Vegetarians had the lowest total energy intake without alcohol, and intermediate values for contribution of macronutrients to total energy intake compared to vegans and meat-eaters (Table 5). Vegans had the lowest mean contribution of total proteins, animal proteins, total lipids, and saturated fatty acids to energy intake and the highest for plant proteins, polyunsaturated fatty acids, total carbohydrates, and simple carbohydrates. Meat-eaters had the lowest contribution of plant proteins, polyunsaturated fatty acids, total carbohydrates, and simple carbohydrates to energy intake and the highest for total proteins, animal proteins, total lipids, and saturated fatty acids (Table 5). The highest proportions of individuals complying with acceptable protein intake was found in vegetarians (Table 6). The highest proportion of individuals whose dietary fiber intake was above 30 g/day was found in vegans. The highest proportion of individuals complying acceptable carbohydrate and lipid intake was found in meat-eaters. The lowest proportion of individuals whose macronutrient intake was within acceptable distribution ranges was found in vegans (Table 6).

### 3.4. Compliance with Recommendations for Micronutrients

The range of prevalence of nutrient inadequacy varied from 0% to 83.4%: 0% iron inadequacy in vegan men as well as 0% phosphorus inadequacy in vegetarian men >65 years of age and in meat-eaters, to 83.4% vitamin B12 inadequacy in vegan women <55 years of age (Table 7). Small variations between diet groups were observed for zinc and phosphorus inadequacies in both sexes, as well as for iron and potassium among men. Men, whatever their diet group, had the lowest iron inadequacy. Vegetarians, compared to other diet groups, had higher prevalence of inadequacy for thiamin (men and women), niacin (only in men <65 years of age), pantothenic acid (close to the prevalence in vegans) and B6 (men <65 years of age and women), zinc (close to the prevalence in vegans), and potassium (for men and women <55 years of age). Vegans had the highest prevalence of inadequacies for total vitamins A, riboflavin (men and women <55 years of age), B12 (for all men, as well as women <55 years of age) and calcium (for women >55 years of age). Meat-eaters had the highest folate, vitamin C (for men <55 years of age and women) and E (for men and women <55 years of age), iron (only in women <55 years of age), and fairly high calcium inadequacies. Vegetarians and vegans also had the lowest vitamins C and E inadequacies. These results were in agreement with mean daily nutrient intakes by age and sex in supplemental Table S1. For example, vegan men also had the lowest mean intake of vitamin B12.

## 4. Discussions

Our study provides new insights into the sociodemographic and nutritional profiles of self-reported vegetarians and vegans in a large observational study. As expected, self-reported vegetarians and vegans were prone to adopt meat substitution strategies such as higher consumption of plant protein-dense products (e.g., soy-based products or legumes). Vegetarians in our study had the most balanced diet and lower prevalence of dietary nutrient inadequacies. 

### 4.1. Sociodemographic Profiles of Vegetarians and Vegans

Vegetarians in our sample were more likely to be women and individuals with higher educational levels, whereas vegans were more likely to be men and individuals with a lower educational level. Moreover, vegetarians and vegans in our study were more likely to belong to lower income categories, as previously reported in a study conducted in Canada [9]. This finding may be explained by the fact that educational level has a higher predictive value than other socioeconomic predictors such as occupation or income. This fact has already been reported and discussed in a previous study from the Nutrinet-Santé cohort [47]. Other previous studies have reported that occupation is related to prestige, skills, and social hierarchy, whereas education can impact skills and knowledge, and thus encourage skills to understand and use all types of nutritional information (guidelines, cooking skills, health promotions messages, etc.) [47]. Thus, this may explain why income and educational level may be associated in different or opposite ways in our study.

Vegetarians and vegans in our study were both younger than meat-eaters, as previously reported in four studies conducted among adults in the UK, Canada, and the U.S. [6,8,9,19]. Income and concerns related to food prices may be a motive to follow a vegetarian diet [48]. In conflict with our findings, it has been reported that vegans were more likely to be manual workers in the UK EPIC-Oxford cohort study [8]. A previous work conducted in the Nutrinet-Santé study indicated that consumption of animal products was higher for manual workers compared to managerial staff [49]. Animal food could have a symbolic role (contribution to physical strength and energy), explaining why vegetarian diets may be less popular in this socioeconomic group [49]. We observed that both vegetarians and vegans were more likely to live alone without children. It has been previously reported that vegans were more likely to be nulliparous, more likely to be single [8,9], and that lacto-vegetarians and strict vegetarians (diet comparable to veganism in our study) were more likely to be married [6]. Indeed, it is possible that individuals switch back to a non-vegetarian diet when they have a child [50,51].

Our findings may also be interpreted in light of sociodemographic determinants of food choice motives such as health, animal welfare, or environment preservation that more deeply established in women [51], who are thus more prone to adopt a vegetarian diet and to reduce meat consumption [9,48]. Moreover, gender representations about meat and masculinity may explain why vegetarianism is more popular among women [52]. Different cultural settings, populations, and different times of investigations [48] could also explain our findings. Indeed, previous studies reported that a reduction of meat consumption was not associated with sociodemographic characteristics such as age or education [48]. It is possible that current growing consumers’ concerns for the protection of the environment may induce dietary changes such the reduction of consumption of animal products [53,54]. The reduction of the consumption of animal products may also concern individuals belonging to lower income categories. Also, unlike a previous study that reported a lower proportion of smokers among vegetarians [8], we did not observe any statistically significant association between smoking status and vegetarianism or veganism in our study sample. It is possible that smoking status is not currently linked with vegetarian diets anymore. 

### 4.2. Compliance with Nutritional Recommendations

The mean contribution of proteins to total energy intake for vegetarians and vegans in our study was similar to those reported in previous studies [6,19]. However, while vegetarians had a higher proportion of individuals with protein intake within acceptable range, they also had a greater proportion of individuals under the acceptable intake range for proteins. A previous study also reported that vegetarians had a higher prevalence of protein inadequacy compared to meat- or fish-eaters [19]. It is recommended to combine proteins from legumes and cereals to reach a higher variety of amino acid intakes [55]. Compared to meat-eaters, vegetarians and vegans had a higher intake of these food groups as well as a higher intake of soy products. Thus, they may consume a high variety of amino acids, as is recommended [1]. The mean contribution of saturated fatty acids to total energy intake was higher and that of polyunsaturated fatty acids were lower [6,19]. Vegetarians and vegans had a similar proportion of individuals within the acceptable distribution range compared to meat-eaters according to the French nutritional recommendations [56]. However, vegans had the highest proportion of individuals under the acceptable distribution range of proteins and lipids, suggesting that within this sample, most vegans had an unbalanced macronutrient intake.

In our study, vegetarians and vegans also had a higher intake of PUFAs than meat-eaters. This may be explained by a greater proportion of pesco-vegetarian diets in our study, in addition to a higher intake of plant sources of these fatty acids [1]. Two studies based on the EPIC-Oxford cohort studies also reported that vegans had the highest intake of PUFAs [8,19]. 

In our study, the majority of vegans had a fiber intake that met French recommendations [57]. A smaller portion of vegetarians and only 10% of meat-eaters met the fiber intake recommendation. Most previous studies also reported higher intakes of fiber for vegans and vegetarians, but the gap between meat-eaters and vegans was even larger in our study. Indeed, the fiber intake was 24 to 41% higher in vegans compared to meat-eaters in previous studies [19,20], whereas it was about 75% higher in our study. 

With regard to micronutrient comparison, vegetarians and vegans had lower intakes of both calcium and vitamin D, in accordance with a previous review [16], especially for women. Additionally, the bioavailability of calcium from plant sources is an issue, especially for vegans [1]. Iron inadequacy was lower in vegetarians and vegans compared to meat-eaters, according to French nutritional recommendations for adults. However, it is likely that vegetarians and especially vegans have very low to null intake of heme iron, respectively. Thus, an issue related to food-specific bioavailability of iron, defined as the extent to which dietary iron is absorbed during digestion and used to maintain normal body functions, may remain. Additionally, a previous study [19] highlighted that iron requirements for vegetarians and vegans may be higher. In fact, a higher consumption of food containing phytates, such as whole grains and legumes [19], or fibers [58] may compromise the absorption of iron for people following a vegetarian diet. Nonetheless, vegetarians and vegans had the lowest vitamin C inadequacy, which may improve iron absorption [59]. Besides, iron deficiency and iron deficiency anemia may not be more common among vegetarians [1,3,14,60]. Similarly, the bioavailability of zinc for vegetarians has raised questions [60,61]. Our results showed that vegans had the highest prevalence of vitamin B12 inadequacy by far. Vitamin B12 deficiency could harm health over a long period (cognitive impairment, stroke, or poor bone health, for example) [1]. However, all of these potential micronutrient inadequacies, such as vitamin B12, may be balanced by the intake of fortified foods and dietary supplements, as is recommended in some cases for these consumers [1]. For example, the prevalence of dietary vitamin B12 inadequacy may have been over-estimated among vegetarians and vegans that take dietary supplements.

Taking into account the whole dietary pattern of individuals using the mPNNS-GS, vegetarians better adhered to French dietary guidelines compared to meat-eaters and vegans. This result is in line with a previous study conducted in Belgium that indicated that different types of vegetarians had a higher Healthy Eating Index mean score compared to meat-eaters [22]. Vegans had a lower mPNNS-GS score, probably due to the computation of the score that allocates points to a moderate consumption of animal products. 

Similar to a previous study conducted in the U.S. [25], the percentage of subjects consuming animal protein substitutes such as soy-based products, cereals, or textured vegetable protein was nearly exclusively consumed by vegetarians and vegans compared to non-vegetarians. The substitution of meat and animal protein by plant-based meat substitutes may contribute to the lower the environmental impact of vegetarian dietary patterns [53,54]. Thus, vegetarian diets are more sustainable [54,62]. 

Some limitations of our study should be acknowledged. First, we used a classification of vegetarianism and veganism based on self-reported food behaviours. A Finnish study [63] highlighted that self-reported vegetarians and vegans differ from operationalized definitions, based on food consumption, of vegetarianism and veganism. Thus, our results may be specific to self-reported vegetarians and vegans and may not be generalizable to all individuals following a vegetarian or vegan diet. Also, we used self-report classification to define diet groups, whereas previous studies used more categories of vegetarianism (differentiation between fish-eaters, vegetarians, and vegans or strict vegetarians). Thus, vegetarians and vegans had low but not null mean intakes of meat and meat products, as well as other animal protein intakes. Self-reported vegetarians or vegans may in fact consume some meat products, seafood, and dairy products. Our results suggest that the use of self-report appears insufficiently accurate to study the relationship between these diets and health outcomes. 

Furthermore, a selection bias is probable, because our sample was based on participants from the NutriNet-Santé study recruited on a voluntary basis with a high proportion of women and participants with a higher educational level. Caution is needed when generalizing the results to the general French population. However, especially for self-reported vegetarians, dietary habits identified in our study may be close to what was reported in previous studies, although other definitions of vegetarianism may have been used, thus improving the external validity of our results.

Comparisons with other studies in terms of nutritional characteristics may be limited by disparities in nutritional recommendations across countries [64] and by the definition of vegetarian diets. Also, dietary supplement intake was not taken into account in the present study. Further investigations assessing whether the intake of dietary supplements could compensate potential dietary inadequacies are requested. To the best of our knowledge, this is the first study on profiles of vegetarians and vegans conducted in France. Accurate information of dietary consumption and nutritional intakes have been collected and analyzed using a validated method [31,32], and taking into account intra-individual variability. Specifically, data were recent and updated and included many “emerging” foods such meat and dairy substitutes.

## 5. Conclusions

In our study, self-reported vegetarians had a better macronutrient composition and overall diet quality, and they may also reach recommendations for critical macronutrients. Also, our results suggest that self-reported vegetarians have higher intake of plant-based, protein-dense foods such as cereals, soy products, or other meat substitutes that were recently added in the French food market. This may help them to maintain a balanced diet. However, issues related to iron and zinc bioavailability and vitamin B12 (especially among vegans) intakes remain, but the intake of food such as meat substitutes and nutrient supplementation needs to be considered. 

Sociodemographic characteristics of self-reported vegetarians and vegans may differ from those of individuals actually following vegetarian or plant-based diets. 

Although such diets may be culturally difficult to accept, at least in some subgroups, well-planned vegetarian dietary patterns could be considered as sustainable diets in light of potential health benefits and a lower environmental impact. Further longitudinal studies are still required to better assess the long-term health effects of vegetarian and vegan diets.

## Figures and Tables

**Table 1:** Sociodemographic characteristics of the sample (Nutrinet-Santé Study 2009–2015, *n* = 93,823).

Sociodemographic Characteristics	*n*	%	*p* ^1^
Sex			<0.0001
Men	20,632	21.9	
Women	73,191	78.1	
Age (years)			<0.0001
18–30	9856	10.5	
30–50	39,082	41.6	
50–65	27,630	29.4	
65+	17,255	18.3	
BMI (kg/m^2^)			<0.0001
≥30	8541	9.1	
25–30	20,059	21.3	
20–25	60,176	64.1	
<20	5047	5.3	
Educational level			<0.0001
Post graduate	32,702	34.8	
Under graduate	29,155	31.0	
Secondary	29,541	31.4	
Primary	2425	2.5	
Occupational categories			
Managerial staff	21,511	22.9	
Intermediate profession	15,171	16.1	
Self-employed	1989	2.1	
Manual worker	18,397	19.6	
Retired	20,949	22.3	
Never employed	15,806	16.8	
Monthly household income classes			<0.0001
Refused to declare	10,951	11.6	
>2700 €	21,752	23.1	
1800–2700 €	21,762	23.1	
1200–1800 €	23,101	24.6	
<1200 €	16,257	17.3	
Smoking status			0.01
Smoker	14,453	15.4	
Ex-smoker	33,296	35.4	
Never smoked	46,074	49.1	
Household composition			<0.0001
Alone without children	21,447	22.8	
Alone with at least one child	4041	4.3	
Two adults living as a couple without children	41,486	44.2	
Two adults living as w couple with at least one child	26,849	28.6	

**^1^** *p* for chi^2^ tests.

**Table 2:** Associations between sociodemographic characteristics and vegetarian and vegan diets using polytomic logistic regression models (reference diet: meat-eaters, Nutrinet-Santé Study 2009–2015, *n* = 93,823).

Sociodemographic Characteristics	Vegetarians		Vegans		Meat-Eaters		Vegetarians vs. Meat-Eaters		Vegans vs. Meat-Eaters		
	(*n* = 2370)		(*n* = 789)		(*n* = 90,664)						
	*n*	%	*n*	%	*n*	%	OR	95% CI	OR	95% CI	*p* ^1^
**Sex**											<0.0001
Women	2015	85.0	595	75.4	70,581	77.9	1.28	(1.13–1.44)	0.65	(0.55–0.77)	
Men	355	15.0	194	24.6	20,083	22.2	1		1		
**Age (years)**											<0.0001
18–30	432	18.2	225	28.5	9199	10.2	1		1		
30–50	1100	46.4	378	47.9	37,604	41.5	1.10	(0.96–1.25)	0.92	(0.76–1.12)	
50–65	601	25.4	132	16.7	26,897	29.7	0.80	(0.70–0.93)	0.32	(0.25–0.41)	
65+	237	10.0	54	6.8	16,964	18.7	0.53	(0.41–0.68)	0.18	(0.11–0.29)	
**BMI (kg/m^2^)**											<0.0001
≥30	119	5.0	55	7.0	8367	9.2	1		1		
25–30	267	11.3	96	12.2	19,696	21.7	1.03	(0.83–1.28)	0.79	(0.56–1.11)	
18.5–25	1664	70.2	521	66.0	57,991	64.0	1.88	(1.55–2.27)	1.18	(0.89–1.58)	
<18.5	320	13.5	117	14.8	4610	5.1	3.75	(3.02–4.67)	2.51	(1.80–3.50)	
**Educational level**											<0.0001
Post graduate	942	39.8	267	33.8	31,493	34.7	1		1		
Under graduate	767	32.4	254	32.2	28,134	31.0	0.93	(0.83–1.03)	1.22	(1.01–1.481)	
Secondary	612	25.8	247	31.3	28,682	31.6	0.75	(0.66–0.84)	1.19	(0.97–1.463)	
Primary	49	2.1	21	2.7	2355	2.6	0.89	(0.66–1.21)	1.74	(1.09–2.794)	
**Occupational categories**											<0.0001
Managerial staff	551	23.3	160	20.3	20,800	22.9	1		1		
Intermediate profession	359	15.2	91	11.5	14,721	16.2	0.92	(0.79–1.06)	0.77	(0.59–1.02)	
Self-employed	69	2.9	34	4.3	1886	2.1	1.65	(1.27–2.15)	2.71	(1.84–4.01)	
Manual worker	479	20.2	177	22.4	17,741	19.6	1.05	(0.91–1.21)	1.12	(0.87–1.44)	
Retired	319	13.5	74	9.4	20,556	22.7	1.04	(0.84–1.29)	1.09	(0.71–1.68)	
Never employed	593	25.0	253	32.1	14,960	16.5	1.30	(1.13–1.49)	1.39	(1.09–1.77)	
**Monthly income (per household unit)**											<0.0001
>2700 €	420	17.7	119	15.1	21,213	23.4	1		1		
Refused to declare	340	14.4	127	16.1	10,484	11.6	1.32	(1.13–1.54)	1.31	(1.00–1.73)	
1800–2700 €	514	21.7	144	18.3	21,104	23.3	1.19	(1.04–1.36)	1.10	(0.85–1.41)	
1200–1800 €	542	22.9	163	20.7	22,396	24.7	1.18	(1.03–1.36)	1.08	(0.83–1.39)	
<1200 €	554	23.4	236	29.9	15,467	17.1	1.49	(1.28–1.73)	1.60	(1.24–2.07)	
**Household composition**											<0.0001
Alone without children	813	34.3	330	41.8	23,677	22.9	1		1		
Alone with at least one child	106	4.5	42	5.3	4489	4.3	0.55	(0.45–0.68)	0.49	(0.35–0.67)	
Two adults living as a couple without children	961	40.6	328	41.6	46,169	44.6	0.72	(0.65–0.80)	0.73	(0.62–0.86)	
Two adults living as a couple with at least one child	490	20.7	89	11.3	29,270	28.3	0.41	(0.36–0.47)	0.18	(0.14–0.24)	

^1^ Odds ratios (95% CI) from the multivariable model including all the following explicative variables: sex, age, BMI, educational level, occupational categories, monthly household income classes, and household composition and adjusted for living area; OR: Odds ratio; CI: Confidence interval.

**Table 3:** Comparisons of mean intakes of food adjusted for sex, age, and total energy intake and mPNNS-GS among vegetarians, vegans, and meat-eaters (Nutrinet-Santé Study 2009–2015, *n* = 93,823).

Food Groups	Vegetarians (*n* = 2370)		Vegans (*n* = 789)		Meat-Eaters (*n* = 90,664)		Vegans vs. Vegetarians	Vegans vs. Meat-Eaters	Vegetarians vs. Meat-Eaters
	Mean ^1^	SEM	Mean ^1^	SEM	Mean ^1^	SEM	*p* ^2^	*p* ^2^	*p* ^2^
Fruit (g)	290.6	3.5	364.2	6.1	245.1	0.7	<0.0001	<0.0001	<0.0001
Vegetables (g)	285.8	2.5	366.0	4.4	216.4	0.5	<0.0001	<0.0001	<0.0001
Legumes (g)	32.8	0.5	73.2	0.9	11.5	0.1	<0.0001	<0.0001	<0.0001
Potatoes and other tubers (g)	45.6	1.0	58.3	1.8	49.0	0.2	<0.0001	<0.0001	<0.0001
Whole starchy food (g)	65.4	1.0	83.4	1.7	33.9	0.2	<0.0001	<0.0001	<0.0001
Refined cereals and starchy foods (g)	127.1	1.7	122.9	2.9	150.3	0.3	0.25	<0.0001	<0.0001
Uncooked cereals and seeds (g)	7.6	0.3	13.0	0.5	1.7	0.1	0.08	<0.0001	<0.0001
Quinoa, corn, and other cereals (g)	16.2	0.4	27.3	0.7	6.6	0.1	<0.0001	<0.0001	<0.0001
Nuts (g)	11.6	0.3	19.6	0.4	4.4	0.0	<0.0001	<0.0001	<0.0001
Oils (g)	11.1	0.2	14.5	0.3	8.9	0.0	<0.0001	<0.0001	<0.0001
Textured soy proteins products (g)	19.7	0.4	61.0	0.6	1.3	0.1	<0.0001	<0.0001	<0.0001
Vegetarian patties (g)	6.9	0.3	12.3	0.5	1.4	0.1	<0.0001	<0.0001	<0.0001
Germinated seeds (g)	7.5	0.3	20.0	0.5	1.9	0.1	<0.0001	<0.0001	<0.0001
Cookies and diet biscuits enriched with cereals ^3^ (g)	4.9	0.3	6.4	0.5	2.1	0.1	0.08	<0.0001	<0.0001
Soy, almond, rice and other plant-based drinks (mL)	160.2	3.1	419.3	5.3	28.5	0.6	<0.0001	<0.0001	<0.0001
Meat (g)	10.0	0.9	10.8	1.5	47.1	0.2	0.05	<0.0001	<0.0001
Offal (g)	1.2	0.3	1.4	0.5	4.1	0.1	0.62	<0.0001	<0.0001
Poultry (g)	6.8	0.7	6.1	1.2	26.8	0.1	0.59	<0.0001	<0.0001
Processed meat (g)	8.8	0.7	5.8	1.1	34.9	0.1	0.02	<0.0001	<0.0001
Fish and other seafood (g)	30.6	0.9	12.8	1.5	39.8	0.2	<0.0001	<0.0001	<0.0001
Eggs (g)	17.3	0.4	5.4	0.8	13.7	0.1	<0.0001	<0.0001	<0.0001
Dairy products (g)	159.2	3.2	45.0	5.5	202.9	0.6	<0.0001	<0.0001	<0.0001
Animal added fats (g)	12.5	0.3	9.5	0.5	13.7	0.1	<0.0001	<0.0001	<0.0001
Salty snacks and biscuits (g)	3.9	0.2	8.1	0.4	4.0	0.0	<0.0001	<0.0001	0.69
Sweet and fatty foods ^4^ (g)	133.3	1.7	93.8	2.9	135.4	0.3	<0.0001	<0.0001	0.05
Drinks, sugary (mL)	40.8	2.2	37.1	3.8	51.1	0.4	0.29	<0.0001	<0.0001
Drinks, sugar-free (mL)	1169.1	11.7	1163.6	20.1	1069.2	2.3	0.29	0.48	0.23
Alcoholic beverages (mL)	107.0	3.1	88.8	5.3	122.0	0.6	0.01	<0.0001	<0.0001
Protein-enriched products (chocolate bars, puddings, etc.) (g)	2.9	0.4	1.2	0.7	1.7	0.1	0.08	0.48	0.01
m-PNNS-GS	7.98	1.63	7.60	1.44	7.82	1.65	<0.0001	<0.0001	<0.0001

^1^ Adjusted mean for age, sex, and total energy intake except for m-PNNS-GS; ^2^ *p* for ANCOVA tests adjusted for age, sex, and total energy intake; ^3^ cookies and diet or digestive biscuits enriched with cereals, rice crackers; ^4^ pastries, biscuits, cookies, chocolate, sweets, honey, jam and sugary spreads; SEM: standard error of the mean, mPNNS-GS: modified Programme National Nutrition Santé Guideline Score.

**Table 4:** Mean nutrient intake adjusted for age and sex among vegetarians, vegans, and meat-eaters (Nutrinet-Santé Study 2009–2015, *n* = 93,823).

Daily Nutrient Intake	Vegetarians (*n* = 2370) ^1^		Vegans (*n* = 789) ^1^		Meat-Eaters (*n* = 90,664) ^1^		Vegans vs. Vegetarians	Vegans vs. Meat-Eaters	Vegetarians vs. Meat-Eaters
	Mean	SEM	Mean	SEM	Mean	SEM	*p* ^2^	*p* ^2^	*p* ^2^
Total Proteins (g)	66.6	0.4	62.0	0.8	80.7	0.1	<0.0001	<0.0001	<0.0001
Plant proteins (g)	33.8	0.4	46.5	0.7	25.7	0.1	<0.0001	<0.0001	<0.0001
Animal proteins (g)	33.9	0.2	15.5	0.3	57.1	0.0	<0.0001	<0.0001	<0.0001
Total lipids (g)	78.3	0.4	72.7	0.7	78.4	0.1	<0.0001	<0.0001	0.14
PUFAs—total (g)	13.3	0.1	17.2	0.2	11.2	0.0	<0.0001	<0.0001	<0.0001
PUFA n3 (g)	1.5	0.0	1.7	0.0	1.3	0.0	<0.0001	<0.0001	0.0022
PUFA n6 (g)	11.2	0.1	15.0	0.2	9.2	0.0	<0.0001	<0.0001	<0.0001
MUFAs (g)	30.1	0.2	30.8	0.4	29.5	0.0	0.30	0.0002	<0.0001
SFAs (g)	29.3	0.2	19.4	0.4	31.8	0.0	<0.0001	<0.0001	<0.0001
Cholesterol (mg)	226.0	3.8	55.4	6.5	305.0	0.7	<0.0001	<0.0001	<0.0001
Total carbohydrates (g)	215.8	0.9	235.7	1.6	199.6	0.2	<0.0001	<0.0001	<0.0001
Simple carbohydrate (g)	99.4	0.7	105.3	1.2	91.8	0.1	0.0003	<0.0001	<0.0001
Fibers (g)	25.9	0.2	34.1	0.3	19.5	0.0	<0.0001	<0.0001	<0.0001
Alcohol (g)	8.0	0.3	6.4	0.5	9.2	0.1	0.0007	<0.0001	<0.0001
Total vitamin A (µg)	1163.1	28.4	1361.3	48.9	1049.4	5.6	<0.0001	<0.0001	0.0070
Thiamin (mg)	1.2	0.0	1.6	0.0	1.2	0.0	0.0004	<0.0001	0.08
Riboflavin (mg)	1.7	0.0	1.7	0.0	1.8	0.0	0.02	<0.0001	0.0006
Niacin (mg)	16.1	0.2	18.2	0.3	19.1	0.0	<0.0001	<0.0001	<0.0001
Pantothenic acid (mg)	5.1	0.0	5.3	0.1	5.3	0.0	0.05	0.10	<0.0001
Vitamin B6 (mg)	1.8	0.0	2.3	0.0	1.8	0.0	<0.0001	<0.0001	0.90
Folate (µg)	394.1	3.0	481.4	5.2	327.2	0.6	<0.0001	<0.0001	<0.0001
Vitamin B12 (µg)	3.6	0.2	2.7	0.3	5.3	0.0	<0.0001	<0.0001	<0.0001
Vitamin C (mg)	130.9	2.4	165.3	4.1	117.0	0.5	<0.0001	<0.0001	<0.0001
Vitamin D (µg)	2.4	0.1	1.9	0.1	2.7	0.0	0.0003	<0.0001	<0.0001
Vitamin E (µg)	14.3	0.1	17.6	0.2	11.3	0.0	<0.0001	<0.0001	<0.0001
Vitamin K	3138.6	7.3	3676.1	12.6	2996.6	1.4	<0.0001	<0.0001	<0.0001
Calcium	960.3	0.1	760.0	0.2	923.5	0.0	<0.0001	<0.0001	<0.0001
Fe (mg)	15.4	2.4	18.6	4.2	13.4	0.5	<0.0001	<0.0001	<0.0001
Mg (mg)	408.1	7.3	495.2	12.6	335.8	1.4	<0.0001	<0.0001	<0.0001
K (mg)	3138.63	17.6	3676.12	30.5	2996.64	2.8	<0.00001	<0.00001	<0.00001
P (mg)	1257.9	7.3	1249.6	12.6	1275.9	1.2	0.36	0.0003	<0.0001
Copper (mg)	2.0	0.0	2.5	0.1	1.7	0.0	<0.0001	<0.0001	<0.0001
Zn (mg)	9.9	0.1	10.0	0.2	10.9	0.0	0.67	<0.0001	<0.0001
Na (mg)	2479.7	20.0	2589.6	34.5	2718.5	3.9	0.01	<0.0001	<0.0001
Manganese (mg)	6.0	0.1	7.7	0.1	4.1	0.0	<0.0001	<0.0001	<0.0001
Iodine (mg)	222.6	5.7	248.3	9.8	180.1	1.1	0.21	<0.0001	<0.0001
Se (mg)	64.5	0.7	64.1	1.3	70.5	0.1	0.66	<0.0001	<0.0001

SEM: Standard error of the mean, PUFA: Poly unsaturated fatty acid, MUFA: Mono unsaturated fatty acid, SFA: Saturated fatty acid; ^1^ adjusted mean for age, sex, and total energy intake (residual method); ^2^ *p* for ANCOVA tests.

**Table 5:** Energy intake and contribution of macronutrients to energy intake among vegetarians, vegans, and meat-eaters (Nutrinet-Santé Study 2009–2015, *n* = 93,823) ^1^.

Energy/Macronutrient Intake	Vegetarians (*n* = 2370)		Vegans (*n* = 789)		Meat-Eaters (*n* = 90,664)		*p* ^2^
	Mean	SD	Mean	SD	Mean	SD	
Total energy intake including alcohol	1814.0	637.0	1877.3	684.02	1898.5	644.0	<0.001
Total energy intake excluding alcohol	1777.4	623.8	1849.4	668.0	1842.7	614.6	<0.001
% of total energy intake excluding alcohol from:							
total proteins	14.2	3.7	12.8	4.3	17.6	4.0	<0.001
plant proteins	7.2	2.2	9.8	3.0	5.4	1.3	<0.001
animal proteins	7.0	4.2	3.0	5.2	12.2	4.3	<0.001
total lipids	38.0	10.0	35.2	11.2	38.5	9.1	<0.001
saturated fatty acids	14.2	5.7	9.6	5.4	15.6	5.2	<0.001
polyunsaturated fatty acids	5.4	3.3	7.1	3.8	4.5	2.6	<0.001
total carbohydrates	47.3	10.0	51.2	12.0	43.3	9.4	<0.001
simple carbohydrates	22.5	8.58	23.6	11.4	20.4	7.4	<0.001

SD: standard deviation. ^1^ adjusted means for age and sex. ^2^ *p* for ANCOVA tests.

**Table 6:** Proportion of individuals under, within, and above acceptable ranges of macronutrient intakes according to the French nutritional recommendations for adults ^1^ among vegetarians, vegans, and meat-eaters (Nutrinet-Santé Study 2009–2015, *n* = 93,823).

Macronutrient Intake	Vegetarians (*n* = 2370)		Vegans (*n* = 789)		Meat-Eaters (*n* = 90,664)		*p* ^2^
	*n*	%	*n*	%	*n*	%	
Proteins ^3^							
Under acceptable distribution range	363	15.3	216	27.3	3686	4.0	<0.0001
Within acceptable distribution range	1751	73.8	509	64.5	62,030	68.4	
Above acceptable distribution range	256	10.8	64	8.1	24,948	27.5	
Total lipids ^4^							
Under acceptable distribution range	857	36.1	380	48.1	30,831	34.0	<0.0001
Within acceptable distribution range	490	20.6	150	19.0	19,544	21.5	
Above acceptable distribution range	1023	43.1	259	32.8	40,289	44.4	
Total carbohydrates ^5^							
Under acceptable distribution range	541	22.8	125	15.8	32,606	35.9	<0.0001
Within acceptable distribution range	918	38.7	242	30.6	37,562	41.4	
Above acceptable distribution range	911	38.4	422	53.4	20,496	22.6	
Fibers ^6^							
<30 g/day	1700	71.7	374	47.4	80,821	89.1	<0.0001
≥30 g/day	670	28.2	415	52.6	9843	10.8	

^1^ Source: ANSES—Agence nationale de sécurité sanitaire de l’alimentation, de l’environnement et du travail (2016) Avis, Actualisation des repères du PNNS: élaboration des références nutritionnelles, rapport d’expertise collective. https://www.anses.fr. ^2^ *p* for chi^2^ tests. ^3^ distribution range for proteins: 10–20% below 70 years of age, 15–20% above 70 years of age. ^4^ distribution range for lipids: 35–40%, below and above 70 years of age. ^5^ distribution range for carbohydrates: 40–55%, below and above 70 years of age. ^6^ distribution range for fibers ≥30 g per day, below and above 70 years of age.

**Table 7:** Prevalence of dietary nutrient inadequacy ^1^ among vegetarians, vegans, and meat-eaters (Nutrinet-Santé Study 2009–2015, *n* = 93,823).

Nutrients	Men (*n* = 20,591)					Women (*n* = 73,191)					
	Vegetarians		Vegans ^2^ *n* = 194	Meat-Eaters		Vegetarians		Vegans		Meat-Eaters	
	<65 Years *n* = 298	>65 Years *n* = 57		<65 Years *n* = 14,230	>65 Years *n* = 5853	<55 Years *n* = 1559	>55 Years *n* = 456	<55 Years *n* = 495	>55 Years *n* = 100	<55 Years *n* = 47,442	>55 Years *n* = 23,139
	%	%	%	%	%	%	%	%	%	%	%
Total vitamin A	10.1	9.3	18.4	12.4	6.6	6.0	1.2	10.4	6.5	5.8	1.2
Thiamin	26.1	22.1	11.1	21.0	24.0	32.1	26.9	16.1	18.1	21.6	21.6
Riboflavin	13.1	11.6	23.8	6.7	5.8	24.7	12.5	35.4	21.9	13.2	8.4
Niacin	11.5	7.0	5.8	1.0	1.0	12.0	6.5	9.1	3.9	26.1	0.5
Pantothenic acid	10.8	5.8	12.5	5.0	3.4	34.5	19.3	36.6	22.5	19.8	12.3
Vitamin B6	23.2	15.7	11.7	18.9	16.1	37.0	23.3	19.8	19.3	27.2	18.4
Folate	3.8	2.9	1.5	13.1	7.9	9.8	3.0	4.6	9.7	17.7	6.8
Vitamin B12	32.8	16.8	69.9	0.8	0.6	45.3	30.9	83.4	49.8	3.7	1.4
Vitamin C	26.9	34.0	18.3	38.8	32.9	41.0	29.4	26.5	24.1	45.7	36.7
Vitamin E	10.2	11.5	1.8	24.0	27.9	28.9	23.0	8.9	26.1	41.6	38.2
Calcium	13.0	43.3	38.2	13.0	49.0	28.1	56.7	60.8	73.9	66.4	64.1
Iron	0.6	0.7	0.0	0.6	0.5	45.2	33.8	24.9	35.3	56.6	46.1
Zinc	9.2	3.1	6.4	1.1	0.3	9.0	10.7	10.7	10.9	0.7	1.4
Magnesium	45.8	49.1	21.9	71.5	69.8	59.7	43.4	36.8	45.3	79.2	68.1
Phosphorus	0.4	0.0	0.5	0.0	0.0	1.4	0.5	5.1	0.6	0.0	0.0
Potassium	12.4	10.8	8.4	10.6	6.0	36.5	17.0	24.1	17.3	32.7	17.7

^1^ The probability of dietary nutrient intakes below the estimated average requirements for the French population ^2^ as only one vegan participant was over 65, a group >65 years of age could not be created.

## Supplementary Materials

The following are available online at www.mdpi.com/2072-6643/9/9/1023/s1, Table S1: Mean nutrient intake by age and sex groups among vegetarians, vegans and meat eaters (Nutrinet-Santé Study 2009–2015, *n* = 93,823).
