# Supplementary material for: Comparison of Sociodemographic and Nutritional Characteristics between Self-Reported Vegetarians, Vegans, and Meat-Eaters from the NutriNet-Santé Study
Source: Nutrients. 2017 Sep 15;9(9):1023. doi: 10.3390/nu9091023 (PMC5622783; doi:10.3390/nu9091023)
Supplement: Supplementary file 1 [file nutrients-09-01023-s001.docx]

Supplemental table 1: Mean nutrient intake by age and sex groups among vegetarians, vegans and meat eaters (Nutrinet-Santé Study 2009-2015, n=93,823)

|  | **Vegetarians** | | | | |  | **Vegans^2^** | | | | |  | **Meat eaters** | | | | |
| --- | --- | --- | --- | --- | --- | --- | --- | --- | --- | --- | --- | --- | --- | --- | --- | --- | --- |
| **Men** (n=20,591) | <65 y | |  | >65 y | |  | n=194 | | | | |  | <65 y | |  | >65 y | |
|  | n=298 | |  | n=57 | |  |  |  |  |  |  |  | n=14,230 | |  | n=5,853 | |
| Daily nutrient intake | Mean | SEM |  | Mean | SEM |  | Mean | |  | SEM | |  | Mean | SEM |  | Mean | SEM |
| Total vitamin A | 1161.2 | 84.1 |  | 1350.7 | 255.1 |  | 1413.5 | |  | 106.8 | |  | 1016.3 | 12.2 |  | 1171.1 | 25.2 |
| Thiamin (µg) | 1.3 | 0.1 |  | 1.4 | 0.2 |  | 1.8 | |  | 0.1 | |  | 1.2 | 0.0 |  | 1.2 | 0.0 |
| Riboflavin (mg) | 1.8 | 0.0 |  | 1.7 | 0.1 |  | 1.7 | |  | 0.1 | |  | 1.8 | 0.0 |  | 1.8 | 0.0 |
| Niacin (mg) | 16.8 | 0.5 |  | 17.9 | 1.1 |  | 19.4 | |  | 0.7 | |  | 19.9 | 0.1 |  | 19.9 | 0.1 |
| Pantothenic acid (mg) | 5.3 | 0.1 |  | 5.5 | 0.2 |  | 5.6 | |  | 0.1 | |  | 5.4 | 0.0 |  | 5.6 | 0.0 |
| Vitamin B6 (mg) | 2.0 | 0.0 |  | 2.0 | 0.1 |  | 2.7 | |  | 0.1 | |  | 1.8 | 0.0 |  | 1.9 | 0.0 |
| Folate (µg) | 424.5 | 9.0 |  | 397.6 | 20.7 |  | 521.4 | |  | 11.4 | |  | 321.7 | 1.3 |  | 352.9 | 2.0 |
| Vitamin B12 (µg) | 3.1 | 0.5 |  | 4.0 | 1.5 |  | 1.4 | |  | 0.7 | |  | 5.4 | 0.1 |  | 6.6 | 0.2 |
| Vitamin C (mg) | 140.0 | 5.9 |  | 112.7 | 11.5 |  | 176.8 | |  | 7.5 | |  | 115.6 | 0.9 |  | 117.8 | 1.1 |
| Vitamin E (µg) | 14.6 | 0.4 |  | 15.8 | 0.8 |  | 18.7 | |  | 0.5 | |  | 10.7 | 0.1 |  | 10.9 | 0.1 |
| Calcium (mg) | 973.0 | 23.6 |  | 949.3 | 47.2 |  | 711.0 | |  | 30.0 | |  | 916.1 | 3.4 |  | 917.1 | 4.7 |
| Iron (mg) | 16.9 | 0.4 |  | 18.9 | 0.9 |  | 20.4 | |  | 0.5 | |  | 13.3 | 0.1 |  | 14.6 | 0.1 |
| Zinc (mg) | 10.0 | 0.3 |  | 10,7 | 0,7 |  | 9.8 | |  | 0.3 | |  | 11.1 | 0.0 |  | 11.7 | 0.1 |
| Magnesium (mg) | 449.7 | 7.8 |  | 449.3 | 17.0 |  | 553.8 | |  | 9.9 | |  | 340.2 | 1.1 |  | 356.6 | 1.7 |
| Phosphorus (mg) | 1305.3 | 22.9 |  | 1355.8 | 47.3 |  | 1259.1 | |  | 29.0 | |  | 1286.4 | 3.3 |  | 1303.7 | 4.7 |
| Potassium (mg) | 3210.1 | 55.2 |  | 3344.0 | 115.3 |  | 3948.8 | |  | 70.1 | |  | 3000.6 | 8.0 |  | 3231.4 | 11.4 |
|  |  |  |  |  |  |  |  |  |  |  |  |  |  |  |  |  |  |
| **Women** (n=73,191) | <55 y | |  | >55 y | |  | <55 y | |  | >55 y | |  | <55 y | |  | >55 y | |
|  | n=1,559 | |  | n=456 | |  | n=495 | |  | n=100 | |  | n=47,442 | |  | n=23,139 | |
| Daily nutrient intake | Mean | SEM |  | Mean | SEM |  | Mean | SEM |  | Mean | SEM |  | Mean | SEM |  | Mean | SEM |
| Total vitamin A | 1117.3 | 29.5 |  | 1213.7 | 72.0 |  | 1248.5 | 52.4 |  | 1394.9 | 153.6 |  | 1008.2 | 5.4 |  | 1201.0 | 10.1 |
| Thiamin (µg) | 1.2 | 0.0 |  | 1.3 | 0.0 |  | 1.6 | 0.0 |  | 1.4 | 0.0 |  | 1.2 | 0.0 |  | 1.2 | 0.0 |
| Riboflavin (mg) | 1.7 | 0.0 |  | 1.8 | 0.0 |  | 1.6 | 0.0 |  | 1.9 | 0.1 |  | 1.7 | 0.0 |  | 1.8 | 0.0 |
| Niacin (mg) | 14.8 | 0.2 |  | 16.9 | 0.3 |  | 16.3 | 0.3 |  | 19.7 | 0.7 |  | 18.2 | 0.0 |  | 19.2 | 0.0 |
| Pantothenic acid (mg) | 4.9 | 0.0 |  | 5.5 | 0.1 |  | 5.0 | 0.1 |  | 5.5 | 0.2 |  | 5.1 | 0.0 |  | 5.5 | 0.0 |
| Vitamin B6 (mg) | 1.7 | 0.0 |  | 1.9 | 0.0 |  | 2.1 | 0.0 |  | 2.0 | 0.1 |  | 1.7 | 0.0 |  | 1.8 | 0.0 |
| Folate (µg) | 374.3 | 3.5 |  | 417.0 | 7.1 |  | 456.7 | 6.3 |  | 436.0 | 15.1 |  | 315.4 | 0.6 |  | 360.8 | 1.0 |
| Vitamin B12 (µg) | 3.1 | 0.2 |  | 4.0 | 0.5 |  | 1.9 | 0.3 |  | 4.9 | 1.0 |  | 4.7 | 0.0 |  | 5.8 | 0.1 |
| Vitamin C (mg) | 126.4 | 3.0 |  | 144.1 | 5.5 |  | 166.3 | 5.4 |  | 138.7 | 11.7 |  | 115.7 | 0.6 |  | 124.2 | 0.8 |
| Vitamin E (µg) | 13.8 | 0.1 |  | 14.7 | 0.3 |  | 17.4 | 0.3 |  | 15.3 | 0.6 |  | 11.5 | 0.0 |  | 12.2 | 0.0 |
| Calcium (mg) | 950.0 | 8.8 |  | 1012.9 | 15.1 |  | 746.7 | 15.7 |  | 893.6 | 32.2 |  | 927.3 | 1.6 |  | 953.6 | 2.1 |
| Iron (mg) | 14.5 | 0.1 |  | 15.6 | 0.3 |  | 17.7 | 0.2 |  | 16.0 | 0.5 |  | 12.9 | 0.0 |  | 14.1 | 0.0 |
| Zinc (mg) | 9.2 | 0.1 |  | 10.3 | 0.2 |  | 9.3 | 0.2 |  | 10.8 | 0.4 |  | 10.5 | 0.0 |  | 11.2 | 0.0 |
| Magnesium (mg) | 381.4 | 2.9 |  | 418.7 | 5.4 |  | 467.0 | 5.1 |  | 420.7 | 11.6 |  | 320.0 | 0.5 |  | 355.4 | 0.8 |
| Phosphorus (mg) | 1205.5 | 8.8 |  | 1315.9 | 15.9 |  | 1200.1 | 15.6 |  | 1303.7 | 33.9 |  | 1249.2 | 1.6 |  | 1306.6 | 2.2 |
| Potassium (mg) | 2960.9 | 21.4 |  | 3314.0 | 39.1 |  | 3442.9 | 37.9 |  | 3417.6 | 83.5 |  | 2867.5 | 3.9 |  | 3188.8 | 5.5 |

^1^as only 1 vegan participant in men was over 65, a group >65y could not be created

^2^adjusted for total energy intake

SEM: Standard Error Model
